# Our New York Letter

**Published:** 1888-01

**Authors:** 


### Correspondence.

### OUR NEW YORK LETTER.

OPIUM SMOKING IN NEW YORK—DR. MORROW'S VIEWS ON THE TREATMENT OF SYPHILIS—HYPODERMIC USE OF MERCURY DISCUSSED—THE NEW YORK CANCER HOSPITAL FORMALLY OPENED—CHEAP DOCTORING IN NEW YORK—THE MUTUAL MEDICAL AID SOCIETY.

*To the Editors Atlanta Medical and Surgical Journal:*

Quite a long chapter might be written upon opium smoking as practiced by white persons in large cities, but it appears that very few people have any accurate knowledge regarding this habit, except the victims themselves, or those who have had some special reason for studying and investigating the subject. In New York city such a thing as smoking the Chinese opium pipe was scarcely heard of a few years ago; but since the Flowery Kingdom has been sending so many representatives to swell our population, this habit has had a steady growth in spite of numerous efforts on the part of our authorities to stamp it out. From the Chinese the habit has spread to a portion of our white community, including mostly young men of the worst reputation, thieves, swindlers of all kinds and a moderately large number of the *demi monde*. For these classes of persons this vice appears to possess a peculiar fascination. They will admit, with apparent pride, being victims to the habit, and talk boastingly of the daily quantity of the drug necessary to satisfy their cravings; but one seldom hears, in New York at least, of a so-called "opium fiend" placing himself under medical treatment for a cure or making any genuine attempt at reform. There are many popular fallacies existing regarding opium smoking and its effects, which certainly are far from true in some, if not most, instances. The preparation of the drug, as used for this purpose, is a syrupy liquid, and, in New York, may be obtained at almost any Chinese store by anybody who asks for it, and at a very moderate cost. To prepare this for the pipe and learn to smoke it requires months of patient practice, and hence it is next to impossible to fall a victim to the habit immediately. The smoker with a long needle dips up a small quantity of this sticky material and subjects it to a peculiar cooking process over a lamp flame, after which it is rolled into a pill and stuck on the pipe-bowl. It is held over the flame again, and while burning the fumes are inhaled. To learn this part of the process thoroughly is said to require about two months of daily practice. Only a short time ago there were numerous resorts or "joints" for smokers here, but most of them are closed at present. It was certainly a strange but not an uncommon sight to see fifty or sixty white persons indulging in this habit in one small apartment, but at the present time these smokers are scattered, yet the habit is by no means on the decline. I have been told that there are over a thousand who practice this habit in New York, not counting the Chinese, and that every week adds about two new victims to the lists. In studying this peculiar mode of dissipation, I have been informed that nearly all beginners experience a certain amount of exhilaration, soon followed by violent and unusually prolonged vomiting, which only wears off after the most persistent use of the pipe. Sleep seldom occurs, or only comes on after smoking several hours. Considerable perseverance is said to be necessary before one can really form the habit, and, on the average, a daily indulgence for a couple of months would be necessary. Judging from observation in New York, there is nothing characteristic about the appearances of opium "fiends," for, while some look pale and poorly nourished, many others appear perfectly healthy. Some never complain of any ailments, but a few are nervous, habitually constipated and eat little. While the practice is continued moderately it does not seem to derange the health much, but when the opium is stopped all admit being made miserable for the want of it. So far as can be learned from those who use opium here in this way, the habit is regarded as injurious, principally from a business point of view, as it consumes so much of the smoker's time that little is left for other occupations.

Dr. P. A. Morrow sometime ago read a paper before the Academy of Medicine on the "Modern method of treating syphilis," in which special reference was made to the use of mercury hypodermically. The writer, after having excellent opportunities for practicing and watching this method under varied circumstances, came to the conclusion that it would never supersede the ordinary way of using mercury, bnt he considered it a valuable agent in syphilis and urged that it should not be disregarded. He believed that where a rapid effect was desired, as when this disease threatened to involve an important organ, we should try to obtain a speedy action in this way. In cases of gastro-intestinal irritation, and also when the patient could not tolerate iodine, he advocated mercury hypodermically. In the discussion following this paper, all admitted that there were many drawbacks in using the drug this way. Dr. E. B. Bronson had treated syphilis by this method, but has discarded it, owing to the pain and accidents following the injections. Dr. R. W. Taylor was in favor of using it in particular cases, his plan being to inject gr. ⅛ of the bichloride with ten drops of water. Dr. L. D. Bulkley was opposed decidedly to the hypodermic use of mercury and did not believe it possessed any advantages whatever. The president, Dr. A. Jacobi, had had unfavorable results following calomel injections and would not resort to them again. He, however, used corrosive sublimate in this way considerably, especially in hereditary syphilis where the early lesions were severe.

The New York Cancer Hospital at 8th avenue and 105 and 106th streets, has been formally opened. The building, which is 180 feet long and 80 feet deep, is four stories high, with three large towers, and will at present accommodate about seventy patients. It is the only institution in the country where nothing but cancer is treated, and at present only females will be admitted, but when sufficient funds are raised, it is intended to erect another building adjoining for men. As a handsome and suitable structure in every respect, it is all that could be desired.

In this city, where medical charity is so much abused and where so many dispensaries already exist, we are to have a Mutual Medical Aid Association. The object of this society is to open thirty-five new dispensaries in different parts of the city, and one has already been started. By paying fifty cents first and afterwards ten cents a week, a man can obtain for himself and family a doctor and the medicine included. A doctor will also call at the home of a patient for twenty-five cents a visit. It seems strange that doctors will not unite and oppose such enterprises which are so unnecessary and yet so injurious to the profession. Common laborers will unite for mutual protection, and why shouldn't physicians ? While we admit that medical charity is so abused in all large cities, we must certainly also admit that the doctors themselves are principally to blame for this state of affairs, and to them we must look for the remedy.

A. F.

*December, 1887.*

